# Occupational Airborne Allergic Contact Dermatitis Caused by a Metal‐Working Fluid Containing Methylisothiazolinone

**DOI:** 10.1111/cod.14728

**Published:** 2024-11-24

**Authors:** Caterina Foti, Lucia Pacello, Benedetta Tirone, Giorgia Sbarra, Riccardo Ravallese, William Andrew Rosato, Nicoletta Cassano, Gino Antonio Vena, Paolo Romita, Piero Lovreglio

**Affiliations:** ^1^ Section of Dermatology, Department of Precision and Regenerative Medicine and Jonian Area University “Aldo Moro” of Bari Bari Italy; ^2^ Interdisciplinary Department of Medicine, Section of Occupational Medicine University “Aldo Moro” of Bari Bari Italy; ^3^ Private Practice Barletta Italy

**Keywords:** airborne allergic contact dermatitis, case report, metal‐working fluid, methylisothiazolinone, occupational allergic contact dermatitis, patch test

Metal‐working fluids (MWFs) are frequent causes of occupational irritant and allergic contact dermatitis (ACD) in metalworkers. MWFs usually contain many additives, such as biocides, emulsifiers, stabilisers, surfactants, lubricants, rust preventives, and others [1]. Various ingredients are important occupational sensitizers, and relevant MWF allergens have been found to be monoethanolamine, resin acids, formaldehyde releasers, and formaldehyde [2].

## 1 Case Report

In June 2024, a 24‐year‐old man was referred for a chronic eczematous dermatitis lasting for 3 years with involvement of the face and neck, sometimes affecting also the forearms. He reported that his dermatitis appeared a few months after being hired as an operator at a computer numerical control (CNC) milling machine and improved significantly during periods of abstention from work. Eczematous lesions spared the periocular region and the hands, which were protected by goggles and gloves, respectively, worn as personal protective equipment (PPE) by the patient during his work activities. In light of the patient‘s history and clinical pattern, we hypothesised the role of occupational exposure to airborne contactants. The patient frequently used a lubricant‐cooling oil (Mecafluid S/2 FF, Petronas Lubricants Italy S.p.A.) at work, sprayed at high pressure on the CNC milling machine components to reduce heat and friction. The CNC milling machine was not equipped with an aspirator, whereas a few suction ducts were present in the rooms where multiple milling machines were allocated. An airborne ACD to the MWF was suspected. Patch tests were performed using the SIDAPA (Società Italiana di Dermatologia Allergologica Professionale e Ambientale) 2023 baseline series (SmartPractice, Rome, Italy srl) occluded for 2 days using allergEAZE patch test chambers (SmartPractice, Phoenix, USA) on Soffix tape (Artsana, Grandate, Italy) [3]. The readings on day (D)2, D4, and D7 revealed a strong positivity to methylisothiazolinone (MI) 0.2% aq. (+++). The patient did not react to methylchlorisothiazolinone (MCI)/MI 0.02% aq. and benzisothiazolinone 0.1% pet., included in the SIDAPA 2023 baseline series. Subsequent patch testing with the MWF 10% pet. showed a positive reaction (++) on D3 and D7. This MWF was found to contain MI and benzisothiazolinone. Unfortunately, other substances present in the MWF (e.g., monoethanolamine, N‐methyldiethanolamine, and iodopropynyl butyl carbamate) were not tested as they are not reported in the list of the testable haptens according to the Italian Medicine Agency AIFA [4]. The eczematous reaction resolved after treatment with topical corticosteroids and avoidance of the exposure to the MWF and any other MI‐containing products.

## 2 Discussion

Isothiazolinones are well‐known contact sensitizers and are preservatives extensively used in a wide range of cosmetic products, as well as household and industrial products, including MWFs [5]. Metal workers are among the occupational groups most frequently sensitised to MI or benzisothiazolinone [6]. Allergic reactions to isothiazolinones contained in MWFs have been described [7, 8, 9].

Airborne ACD from isothiazolinone derivatives is frequently associated with the use of paints and can sometimes occur with exposure to detergents [5], whereas occupational airborne ACD to isothiazolinones from other sources appears to be uncommon. Interestingly, Özkaya et al. described a case of contact allergy to MCI/MI in a maintenance and repair tramline worker with different clinical manifestations, including an airborne ACD caused by the exposure to rust remover and surface cleaner aerosols containing MCI/MI [9].

Given our patient‘s experience, we suggest considering the possibility of airborne ACD in individuals who are exposed to sprayed or vaporised MWFs containing isothiazolinone derivatives. This report highlights the need for increased awareness, more efficient PPE, and enhanced training of workers in order to prevent occupational contact dermatitis.

## Author Contributions

**Caterina Foti:** conceptualization, methodology, supervision. **Lucia Pacello:** conceptualization, writing – original draft. **Benedetta Tirone:** conceptualization, writing – original draft. **Giorgia Sbarra:** investigation, writing – original draft. **Riccardo Ravallese:** investigation, methodology. **William Andrew Rosato:** methodology, writing – original draft. **Nicoletta Cassano:** conceptualization, writing – review and editing, supervision. **Gino Antonio Vena:** conceptualization, writing – review and editing, supervision. **Paolo Romita:** conceptualization, writing – review and editing, supervision. **Piero Lovreglio:** writing – original draft, supervision, methodology.

## Consent

The patient has signed written formal consent to publication of both medical history and clinical pictures.

## Conflicts of Interest

The authors declare no conflicts of interest.
